# Supplementary material for: The Molecular Epidemiology of HIV-1 in Russia, 1987–2023: Subtypes, Transmission Networks and Phylogenetic Story
Source: Pathogens. 2025 Jul 26;14(8):738. doi: 10.3390/pathogens14080738 (PMC12388890; doi:10.3390/pathogens14080738)
Supplement: Supplementary file 1 [file pathogens-14-00738-s001.zip › Supplementary Figure S1.pdf]

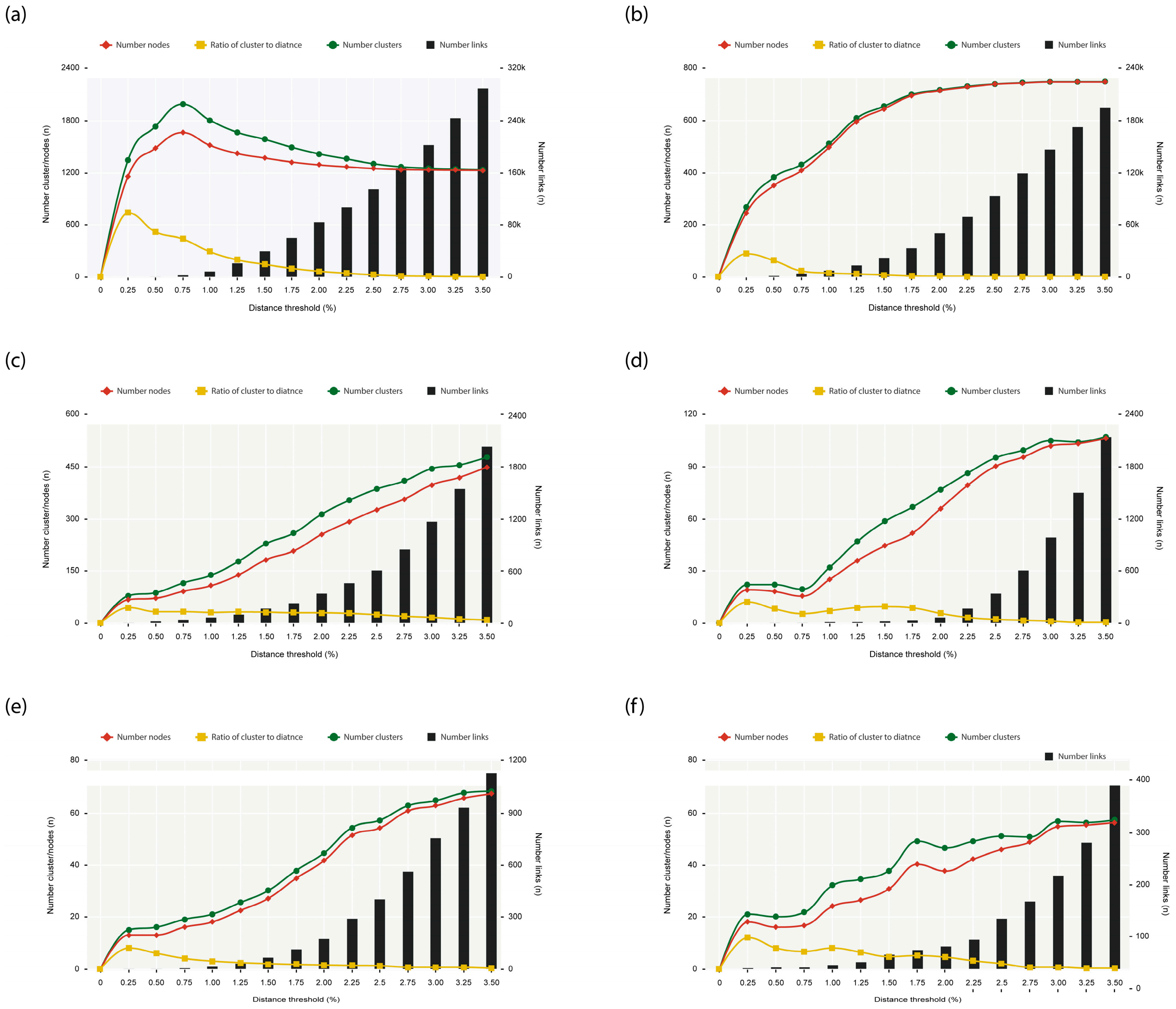

Figure S1. Selection of the optimal distance threshold for the various HIV-1 subtypes. (**a**) subtype A6; (**b**) 63_02A6; (**c**) subtype B; (**d**) 02_AGFSU; (**e**) 03_A6B; (**f**)14/73_BG. Curves represented the number of clusters, the number of nodes or the ratio of the number of clusters to distance at different distance thresholds, as indicated in the legend; bar represented the number of links.
