# Supplementary material for: The Molecular Epidemiology of HIV-1 in Russia, 1987–2023: Subtypes, Transmission Networks and Phylogenetic Story
Source: Pathogens. 2025 Jul 26;14(8):738. doi: 10.3390/pathogens14080738 (PMC12388890; doi:10.3390/pathogens14080738)
Supplement: Supplementary file 1 [file pathogens-14-00738-s001.zip › Supplementary Figure S2.pdf]

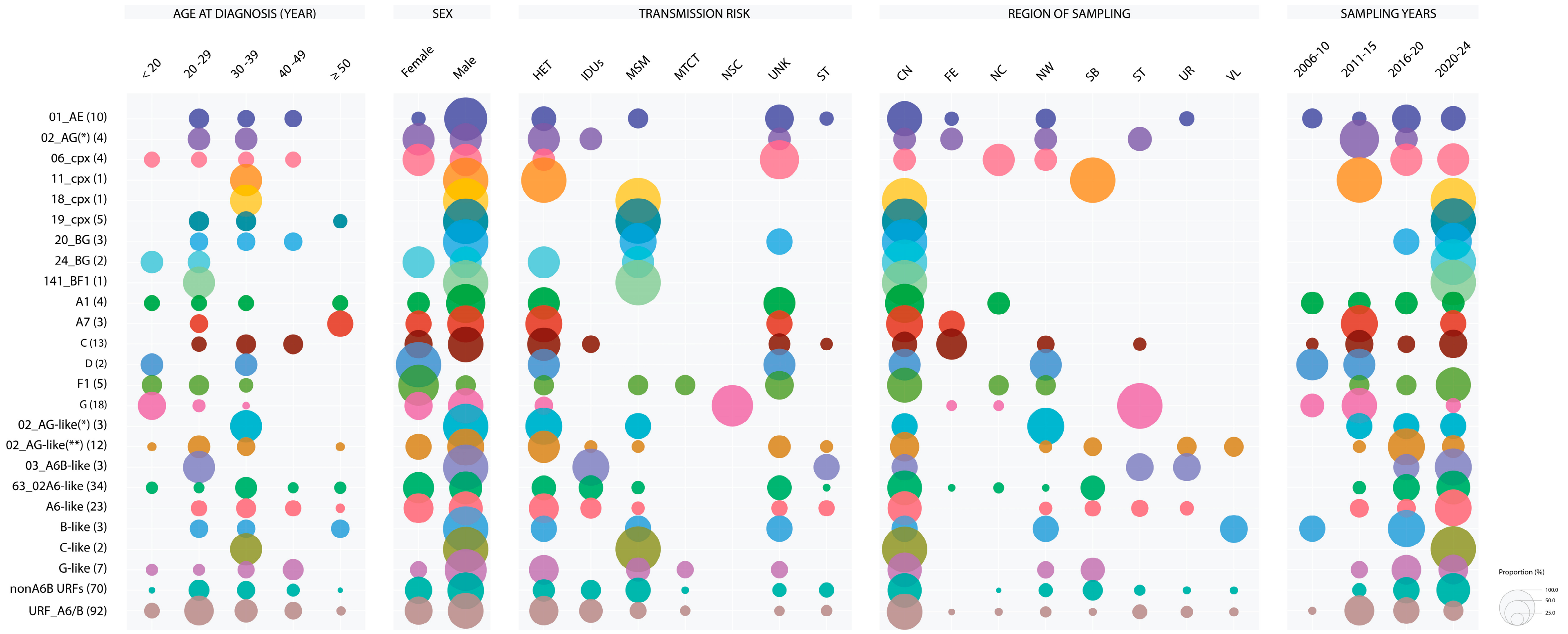

Figure S2. General characteristics of study participants stratified on HIV-1 subtypes of category "others". The proportion for each category is displayed in bubble as described in the legend. The bubble is colored by subtype. Numbers in parentheses indicate the number of cases. The 02_AG$_{FSU}$ are marked with a star and the 02_AG$_{African}$ have two stars. HET, heterosexual contacts; IDUs, injecting drug users; MSM, men who have sex with men; MTCT, mother-to-child transmission; NSC, nosocomial transmission; UNK, unknown; ST, sexual transmission (without specification); CN, Central Federal District (FD); FE, Far East FD; NC, North Caucasian FD; NW, Northwestern FD; SB, Siberian FD; ST, Southern FD; UR, Ural FD; VL, Volga FD.
