# Supplementary material for: The Molecular Epidemiology of HIV-1 in Russia, 1987–2023: Subtypes, Transmission Networks and Phylogenetic Story
Source: Pathogens. 2025 Jul 26;14(8):738. doi: 10.3390/pathogens14080738 (PMC12388890; doi:10.3390/pathogens14080738)
Supplement: Supplementary file 1 [file pathogens-14-00738-s001.zip › Supplementary Figure S3.pdf]

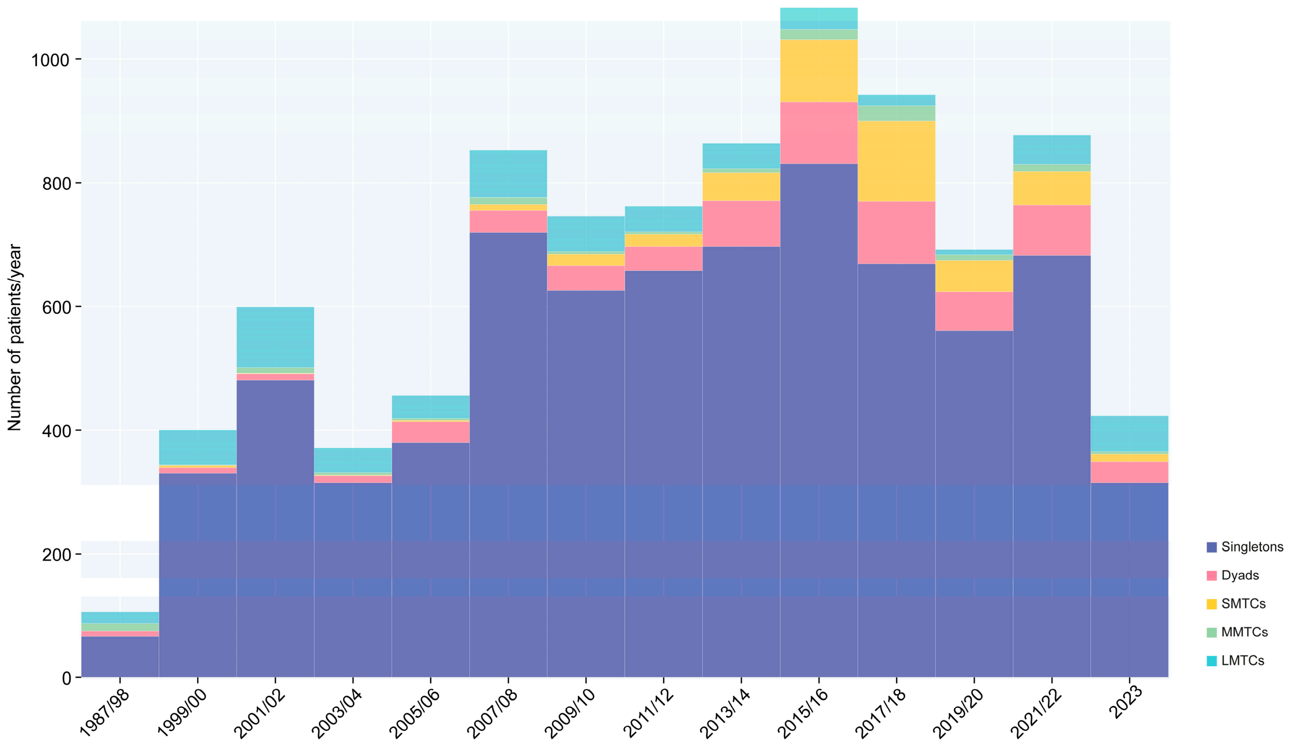

Figure S3. Temporal trend for membership in molecular transmissions clusters (MTCs) of different types (sizes). The x-axis represents the midpoint of the 2-year in which study patients have been diagnosed with HIV infection. LMTCs, large MTCs; MMTCs, medium MTCs; SMTCs, small MTCs.
