# Supplementary material for: The Molecular Epidemiology of HIV-1 in Russia, 1987–2023: Subtypes, Transmission Networks and Phylogenetic Story
Source: Pathogens. 2025 Jul 26;14(8):738. doi: 10.3390/pathogens14080738 (PMC12388890; doi:10.3390/pathogens14080738)
Supplement: Supplementary file 1 [file pathogens-14-00738-s001.zip › Supplementary Figure S4.pdf]

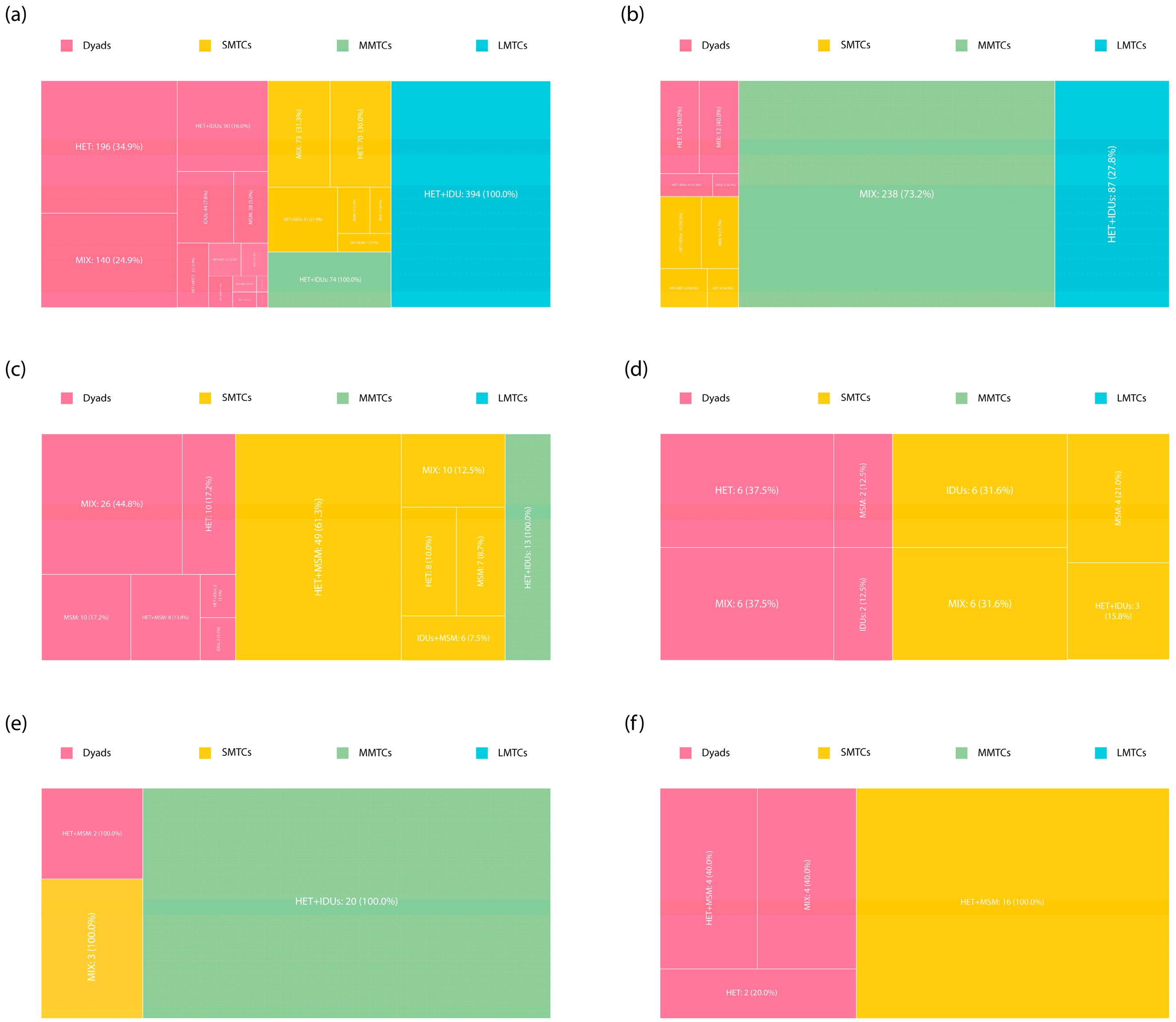

Figure S4. TreeMap depicting distribution of transmission risk within the identified types of molecular transmissions clusters (MTCs) among the various HIV-1 subtypes. (**a**) subtype A6; (**b**) 63_02A6; (**c**) subtype B; (**d**) 02_AGFSU; (**e**) 03_A6B; (**f**) 14/73_BG. MTCs were categorized based on their size, including singletons (consisting of one sequence) and dyads (consisting of 2 sequences), small MTCs (SMTCs, consisting of 2–6 sequences), medium MTCs (MMTCs, consisting of 10–20 sequences) and large MTCs (LMTCs, containing more than 20 sequences). The figure inside the rectangle corresponds to the number (%) of membership in MTC. The rectangles size is proportional to the share of the cell contribution. HET, heterosexual contacts; IDUs, injecting drug users; MSM, men who have sex with men; MTCT, mother-to-child transmission; NSC, nosocomial transmission. MTCs including at least one member with an unknown and/or sexual transmission (without specification) are marked as "Other".
