# Supplementary material for: The Molecular Epidemiology of HIV-1 in Russia, 1987–2023: Subtypes, Transmission Networks and Phylogenetic Story
Source: Pathogens. 2025 Jul 26;14(8):738. doi: 10.3390/pathogens14080738 (PMC12388890; doi:10.3390/pathogens14080738)
Supplement: Supplementary file 1 [file pathogens-14-00738-s001.zip › Supplementary Figure S5.pdf]

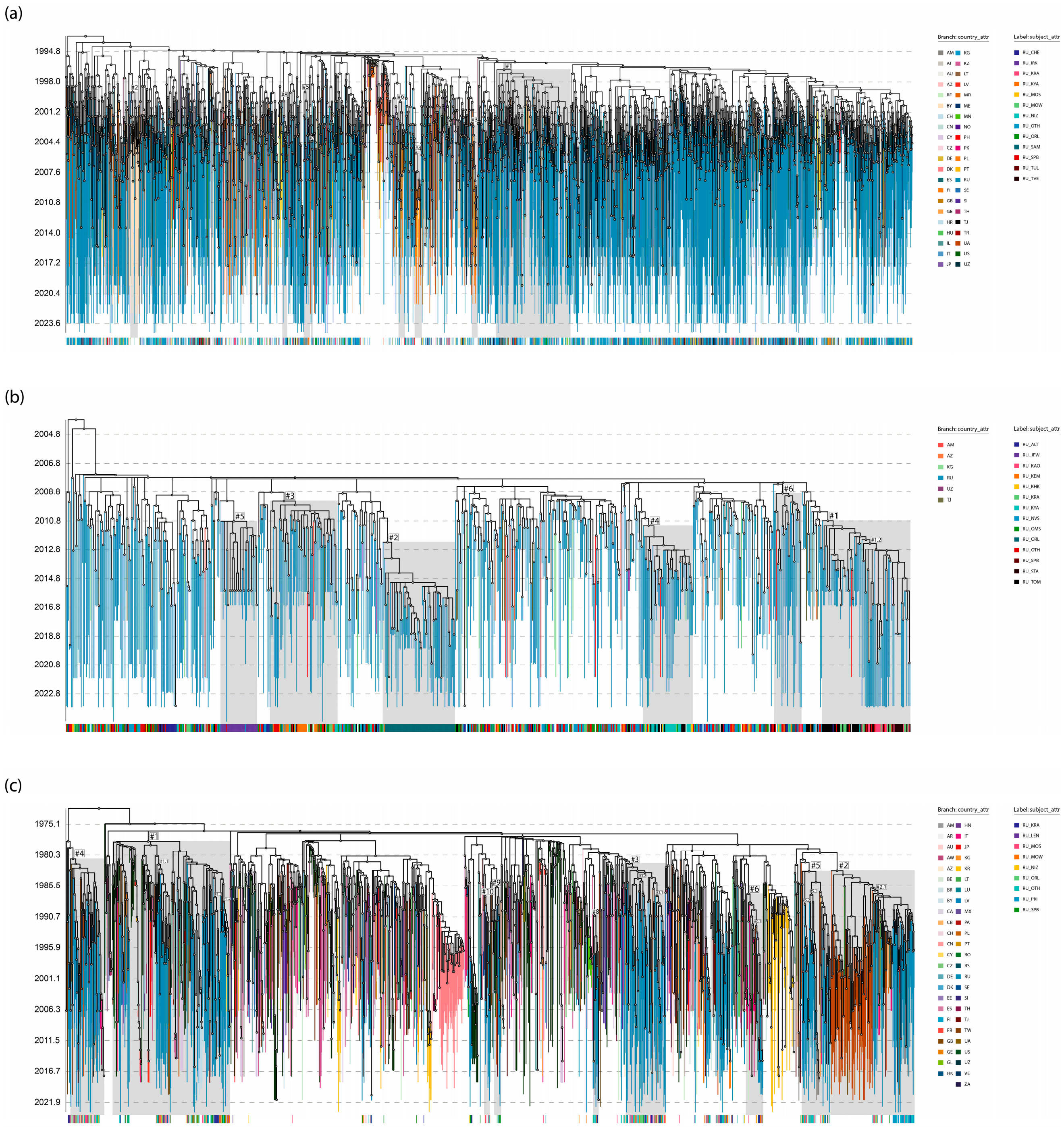

(a)
1994.8
1998.0
2001.2
2004.4
2007.6
2010.8
2014.0
2017.2
2020.4
2023.6
Branch: country_attr
Label: subject_attr
(b)
2004.8
2006.8
2008.8
2010.8
2012.8
2014.8
2016.8
2018.8
2020.8
2022.8
#1
#2
#3
#4
#5
#6
Branch: country_attr
Label: subject_attr
(c)
1975.1
1980.3
1985.5
1990.7
1995.9
2001.1
2006.3
2011.5
2016.7
2021.9
#1
#2
#3
#4
#5
#6
Branch: country_attr
Label: subject_attr

(d)

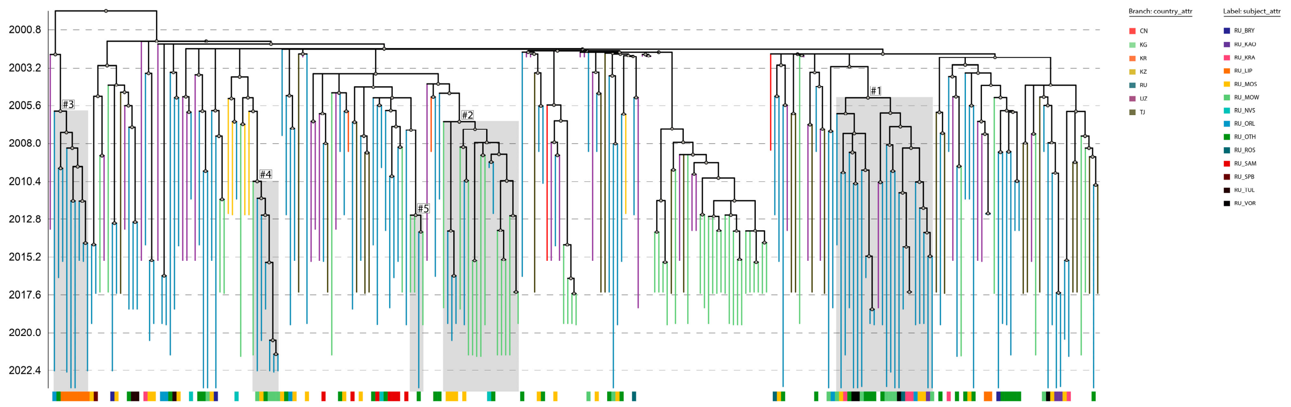

(e)

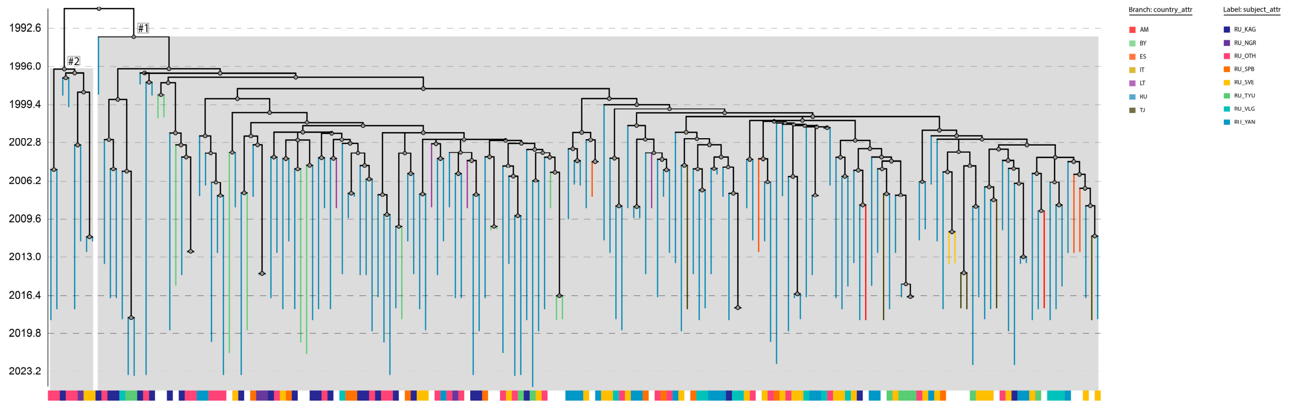

(f)

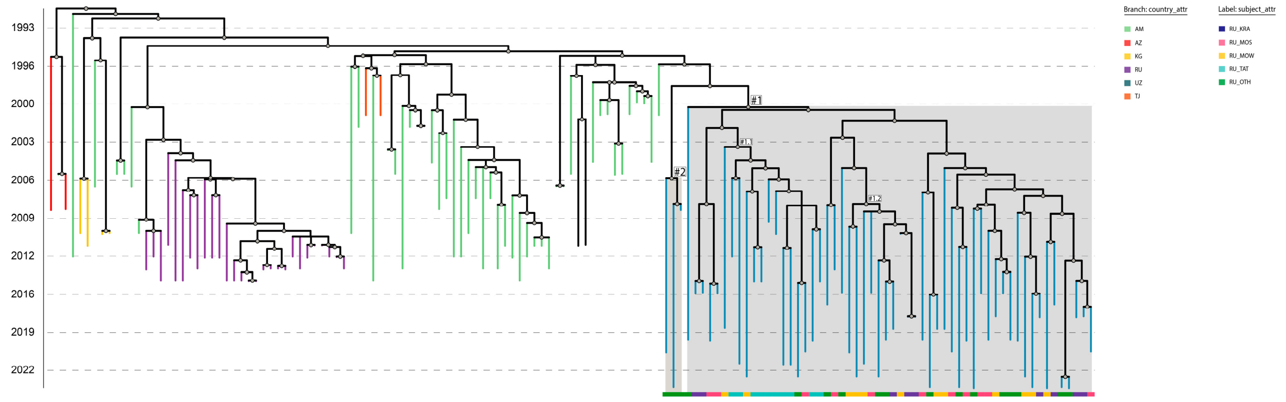

Figure S5. Nextstrain maximum-likelihood (ML) phylogeny of Russian HIV-1 sequences with publicly available global sequences. ML-tree shows a time calibrated phylogenies and revealing similarities of most closely related to Russia global sequences from a BLAST search at GenBank for HIV-1 subtype: (**a**) subtype A6; (**b**) 63_02A6; (**c**) subtype B; (**d**) 02_AG$_{FSU}$; (**e**) 03_A6B; (**f**) 14/73_BG. Branches are colored by country of sampling as indicated in the legends. Branch lengths of ML tree are drawn to scale with the dotted lines indicating calendar years. The grey areas indicate the positions of major clusters (indicated as #1…*n*) identified in the region. The trees were rooted through HIV-1 subtype J. The graphics were generated using the auspice (https://auspice.us, accessed on 11 February 2025) and iTOL (https://itol.embl.de, accessed on 11 February 2025).
