# Supplementary material for: The Molecular Epidemiology of HIV-1 in Russia, 1987–2023: Subtypes, Transmission Networks and Phylogenetic Story
Source: Pathogens. 2025 Jul 26;14(8):738. doi: 10.3390/pathogens14080738 (PMC12388890; doi:10.3390/pathogens14080738)
Supplement: Supplementary file 1 [file pathogens-14-00738-s001.zip › Supplementary Tables.pdf]

Table S1. Root-to-tip regression analysis of the phylogenetic temporal signal on our HIV-1 sequences datasets.

Subtype Dataset [1]	Sequences Number, *n* [2]	Sampling Year	Correlation Coefficient, *r* [3]	Determination Coefficient, r^2 [3]
A6				
#1	12396 (17‡ + 7659†)	1997–2024	0.479	0.229
#2	3127 (15‡ + 2159†)	1997–2024	0.553	0.306
63_02A6				
#1	1372 (572‡ + 747†)	2008–2024	0.581	0.338
#2	793 (276‡ + 468†)	2008–2024	0.604	0.365
B				
#1	3034 (0‡ + 531†)	1978–2024	0.597	0.356
#2	1760 (0‡ + 449†)	1978–2023	0.568	0.323
02_AG$_{FSU}$				
#1	309 (18‡ + 111†)	2002–2023	0.565	0.319
#2	254 (18‡ + 108†)	2002–2023	0.592	0.350
03_A6B				
#1	300 (197‡ + 68†)	1997–2023	0.589	0.347
#2	177 (87‡ + 59†)	1997–2023	0.616	0.379
14/73_BG				
#1	143 (0‡ + 59†)	1999–2023	0.759	0.576
#2	143 (0‡ + 59†)	1999–2023	0.759	0.576

[1] The initial dataset of sequences (#1) and grouped (uniform) sampling (#2) formed for evolutionary and demographic reconstruction (See pp. 2.5 Materials and Methods); [2] Indicates the overall number of sequences in dataset; the figures in parentheses indicate the number of Russian sequences obtained from the NCBI GenBank using the BLAST search (‡) and/or produced in this study (†); [3] The root-to-tip genetic distance against sampling time are shown for ML phylogenies were inferred with IQ-TREE with 1000 replicates for bootstrap under the GTR+I+G model of nucleotide substitution. The correlation and determination coefficient was estimated by TempEst v1.5.

Table S2. The sampling date for the HIV-1 sequences used in this study.

	**HIV-1 Subtype**							
	**Total (N = 9500, 100.0%)**	**A6 (N = 7659; 80.6%)**	**63_02A6 (N = 747; 7.9%)**	**B (N = 531; 5.6%)**	**02_AG$_{FSU}$ (N = 111; 1.2%)**	**03_A6B (N = 68; 0.7%)**	**14/73_BG (N = 59; 0.6%)**	**Others [1] (N = 325; 3.4%)**
before 2000	1 (0)	0 (0)	0 (0)	1 (0.2)	0 (0)	0 (0)	0 (0)	0 (0)
2001–2005	3 (0)	2 (0)	0 (0)	1 (0.2)	0 (0)	0 (0)	0 (0)	0 (0)
2006–2010	582 (6.1)	481 (6.3)	1 (0.1)	62 (11.7)	5 (4.5)	16 (23.5)	4 (6.8)	13 (4.0)
2011–2015	2473 (26.1)	2076 (27.1)	102 (13.7)	150 (28.2)	25 (22.5)	24 (35.3)	12 (20.3)	84 (25.8)
2016–2020	3652 (38.4)	3045 (39.8)	227 (30.4)	197 (37.1)	41 (36.9)	19 (27.9)	26 (44.1)	97 (25.8)
after 2020	2789 (29.4)	2055 (26.8)	417 (55.8)	120 (22.6)	40 (36.0)	9 (13.2)	17 (28.8)	131 (40.4)

[1] 01_AE (*n* = 10), 02_AG$_{African}$ (*n* = 4), 06_cpx (*n* = 4), 11_cpx (*n* = 1), 18_cpx (*n* = 1), 19_cpx (*n* = 5), 20_BG (*n* = 3), 24_BG (*n* = 2), 141_BF1 (*n* = 1), A1 (*n* = 4), A7 (*n* = 3), C (*n* = 13), D (*n* = 2), F1 (*n* = 5), G (*n* = 18), URF_A6B (*n* = 92), non-A6B URFs (*n* = 70), 02_AG$_{African}$-like (*n* = 3), 02_AGFSU-like (*n* = 12), 03_A6B-like (3), 63_02A6-like (*n* = 34), A6-like (*n* = 23), B-like (*n* = 3), C-like (*n* = 2) and G-like (*n* = 7). See Figure S2 for a more complete description of these subtypes data.

Table S3. Characteristics of the different HIV-1 molecular transmission clusters (MTCs) in Russia.

Subtype	Molecular Transmission Cluster (name)	Tips, *N*	Sex		Transmission risk							Median Age at Diagnosis (Year, IQR)	Region of Sampling (at the Level of Subject) ^{1}	Sampling Year
			Male	Female	HET	IDUs	MSM	MTCT	NSC	ST	UNK			
A6	0l394	394	213	181	127	186	2	12	1	65	1	24.0 (20.0–30.0)	alt^{1}, amu^{1}, bas^{9}, bry^{1}, cha^{4}, ^{che8}, irk^{43}, iva^{1}, kal^{5}, kem^{3}, kha^{22}, kir^{1}, kra^{3}, kug^{1}, kya^{23}, len^{1}, lip^{1}, mos^{82}, mow^{4}, niz^{6}, nvs^{1}, oms^{1}, ore^{2}, orl^{18}, row^{3}, rya^{11}, sak^{1}, sam^{31}, sar^{29}, smo^{3}, spb^{1}, sta^{1}, sve^{20}, tat^{7}, tul^{2}, tyu^{1}, udm^{1}, uly^{5}, vgg^{26}, vor^{4}, yan^{4}, yar^{2}	2006–2023
	104q	4	2	2	4	0	0	0	0	0	0	23.5 (22.0–25.0)	kra^{4}	2014
	106t	3	0	3	3	0	0	0	0	0	0	22.0 (19.0–25.0)	kra^{3}	2013–2018
	112t	3	3	0	1	0	2	0	0	0	0	27.0 (26.0–35.0)	mow^{3}	2018–2021
	114t	3	2	1	3	0	0	0	0	0	0	39.0 (32.0–39.0)	kra^{3}	2016
	126t	3	2	1	2	1	0	0	0	0	0	39.0 (34.0–44.0)	lip^{3}	2012
	142q	4	0	4	4	0	0	0	0	0	0	22.0 (21.0–30.5)	mos^{4}	2013–2014
	145t	3	1	2	3	0	0	0	0	0	0	33.0 (20.0–42.0)	kra^{3}	2012–2019
	146l10	10	7	3	3	7	0	0	0	0	0	24.5 (22.0–32.0)	kya^{10}	2012–2016
	148q	4	4	0	1	0	1	0	1	0	1	32.0 (30.0–39.0)	mos^{2}, mow^{2}	2018–2019
	157t	3	3	0	2	0	0	0	1	0	0	42.0 (3.0–42.0)	kra^{3}	2014–2022
	159t	3	2	1	3	0	0	0	0	0	0	30.0 (29.0–42.0)	kra^{3}	2017
	161t	3	3	0	0	0	3	0	0	0	0	26.0 (22.0–26.0)	mow^{2}, kra^{1}	2015–2018
	164q	4	4	0	0	0	4	0	0	0	0	27.0 (23.0–27.5)	kra^{4}	2017
	167t	3	1	2	2	1	0	0	0	0	0	31.0 (24.0–34.0)	orl^{3}	2018–2021
	193t	3	2	1	2	1	0	0	0	0	0	42.0 (30.0–54.0)	che^{2},tat^{1}	2021
	198t	3	1	2	2	0	0	0	0	0	1	41.0 (26.0–45.0)	bry^{1},or^{l2}	2022–2023
	201t	3	2	1	1	2	0	0	0	0	0	34.0 (30.0–35.0)	orl^{3}	2018–2021
	202q	4	3	1	2	0	0	0	0	0	2	39.5 (35.5–42.5)	orl^{4}	2021–2023
	203l5	5	5	0	1	2	0	0	0	1	1	36.0 (33.0–40.0)	orl^{5}	2019–2023
	206q	4	2	2	3	0	0	0	0	0	1	42.0 (37.5–50.5)	orl^{3}, lip^{1}	2018–2019
	209l5	5	3	2	1	3	0	1	0	0	0	35.0 (24.0–36.0)	orl^{5}	2017–2018
	210t	3	2	1	3	0	0	0	0	0	0	30.0 (29.0–31.0)	orl^{3}	2018
	213q	4	1	3	3	1	0	0	0	0	0	43.5 (34.0–54.5)	orl^{3}, kur^{1}	2018–2022
	214l5	5	3	2	4	1	0	0	0	0	0	34.0 (30.0–35.0)	orl^{5}	2018–2019
	216t	3	2	1	3	0	0	0	0	0	0	45.0 (29.0–49.0)	orl^{3}	2018–2019
	222q	4	3	1	1	1	0	0	0	0	2	34.0 (30.0–38.0)	orl^{4}	2018–2023
	224l6	6	3	3	3	3	0	0	0	0	0	32.0 (26.0–38.0)	orl^{6}	2018–2021
	231t	3	3	0	0	0	3	0	0	0	0	25.0 (21.0–25.0)	mow^{3}	2022
	236t	3	1	2	3	0	0	0	0	0	0	32.0 (32.0–39.0)	kra^{3}	2015–2017
	241l7	7	3	4	1	4	0	0	0	2	0	30.0 (29.0–37.0)	lip^{7}	2014–2015
	243q	4	2	2	0	4	0	0	0	0	0	26.5 (24.0–29.0)	lip^{4}	2015–2022
	257l12	12	7	5	2	7	0	0	0	1	2	37.0 (31.5–38.5)	orl^{12}	2018–2022
	258l9	9	8	1	2	5	0	0	0	0	2	39.0 (32.0–39.0)	orl^{9}	2018–2023
	259l7	7	4	3	3	2	0	0	0	0	2	37.0 (30.0–47.0)	orl^{7}	2018–2023
	262l20	20	13	7	8	10	0	0	0	1	1	36.0 (33.0–44.0)	orl^{20}	2018–2023
	273q	4	4	0	2	0	2	0	0	0	0	29.0 (23.5–32.0)	kra^{5}	2016–2019
	275q	4	4	0	1	0	3	0	0	0	0	27.5 (21.0–35.0)	kra^{2}, tve^{1}, mow^{1}	2017–2019
	280t	3	1	2	3	0	0	0	0	0	0	32.0 (27.0–32.0)	kra^{3}	2017
	284t	3	2	1	2	0	0	1	0	0	0	18.0 (0–31.0)	orl^{3}	2018–2021
	28q	4	4	0	4	0	0	0	0	0	0	28.5 (20.0–38.5)	sak^{3}, kha^{1}	2012–2013
	292t	3	3	0	0	0	0	0	0	1	2	30.0 (30.0–44.0)	kha^{1}, mow^{1}, orl^{1}	2018–2022
	294t	3	1	2	3	0	0	0	0	0	0	34.0 (29.0–55.0)	mos^{2}, cha^{1}	2018–2023
	302l6	6	1	5	5	0	0	0	0	0	1	33.5 (25.0–41.0)	orl^{6}	2018–2022
	306q	4	3	1	2	2	0	0	0	0	0	34.0 (31.0–36.5)	orl^{4}	2018–2021
	309l5	5	3	2	4	1	0	0	0	0	0	29.0 (27.0–51.0)	orl^{5}	2018–2023
	30t	3	3	0	0	0	0	0	0	3	0	2.0 (2.0–25.0)	kem^{3}	2014–2016
	315l9	9	3	6	4	0	0	2	0	3	0	29.0 (19.0–34.0)	kra^{9}	2014–2023
	316l6	6	3	3	4	2	0	0	0	0	0	23.5 (23.0–24.0)	kra^{5}, cha^{1}	2017–2023
	321t	3	2	1	2	1	0	0	0	0	0	34.0 (34.0–40.0)	orl^{3}	2018–2022
	322t	3	1	2	2	1	0	0	0	0	0	47.0 (27.0–49.0)	orl^{3}	2018–2022
	324t	3	3	0	1	0	0	0	0	0	2	20.0 (19.0–38.0)	mow^{2}, mos^{1}	2017–2020
	326t	3	2	1	1	2	0	0	0	0	0	23.0 (22.0–31.0)	orl^{3}	2018
	32t	3	0	3	3	0	0	0	0	0	0	32.0 (32.0–32.0)	kra^{3}	2015–2016
	43q	4	1	3	3	1	0	0	0	0	0	31.0 (28.5–37.0)	orl^{4}	2021–2022
	47q	4	3	1	1	3	0	0	0	0	0	32.0 (29.0–33.5)	orl^{4}	2018–2019
	67l13	13	5	8	7	4	0	0	0	0	2	34.0 (30.0–39.0)	orl^{13}	2018–2023
	68l19	19	14	5	4	14	0	0	0	0	1	31.0 (28.0–38.0)	orl1^{8}, mos^{1}	2018–2023
	6t	3	3	0	0	3	0	0	0	0	0	26.0 (23.0–42.0)	niz^{3}	2012–2023
	71t	3	1	2	0	0	0	0	2	1	0	5.0 (2.0–30.0)	tve^{3}	2016
	74t	3	3	0	0	0	3	0	0	0	0	26.0 (18.0–26.0)	kra^{3}	2016–2017
	84t	3	3	0	1	1	0	0	1	0	0	26.0 (26.0–72.0)	smo^{3}	2014
	89q	4	2	2	4	0	0	0	0	0	0	51.0 (51.0–51.0)	mos^{4}	2019
	97t	3	2	1	0	0	0	2	0	1	0	4.0 (3.0–5.0)	cha^{3}	2023
	9t	3	1	2	1	2	0	0	0	0	0	26.0 (23.0–54.0)	kha^{3}	2012
	272t	3	3	0	2	0	1	0	0	0	0	24.0 (22.0–24.0)	kra^{3}	2017–2021
	Dyads	522	282	240	281	104	37	23	15	43	19	31.0 (24.0–39.0)	alt^{6}, amu^{2}, ark^{3}, bry^{9}, bur^{2}, cha^{17},	2007–2023

													che[16], irk[14], iva[1], jew[2], kao[7], kar[2], kem[5], kha[6], khk[3], kra[115], kug[8], kur[5], kya[10], len[3], lip[46], mag[3], mos[39], mow[18], niz[1], nvs[3], oms[2], ore[2], orl[93], per[2], pri[2], psk[2], ros[2], rya[3], sah[2], sak[4], sam[16], sar[4], spb[8], sta[1], tat[6], tul[2], tve[5], tyu[3], udm[2], vlg[4], vor[3], yan[8]	
	Singletons	6436	3665	2771	2931	1813	165	235	20	864	408	30.0 (23.0–38.0)	alt[86], amu[10], ark[10], ast[2], bas[37], bel[2], bry[82], bur[48], cha[96], che[258], crm[2], dag[1], ing[8], irk[101], iva[17], jew[23], kab[3], kag[3], kai[2], kal[39], kao[27], kar[15], kem[68], kha[48], khk[45], khm[19], kir[4], kos[20], kra[424], kug[57], kur[41], kya[217], len[52], lip[68], mag[26], mor[15], mos[1038], mow[167], mur[20], ngr[3], niz[235], nvs[25], oms[26], ore[44], orl[608], per[39], pnz[21], pri[18], ros[45], rya[77], sah[17], sak[55], sam[269], sar[65], sev[1], smo[44], spb[565], sta[13], sve[120], tat[108], tam24, tul[187], tve[176], tyu[59], udm[45], uly[13], vgg[41], vla[3], vlg[9], vor[83], yan[97], yar[70], zab[28], tom[2]	2002–2024
	Overall	7659	4351	3308	3484	2195	226	276	41	986	451	30.0 (23.0–37.0)	-	
63_02A6	0l87	87	58	29	31	50	0	0	0	2	4	35.0 (29.0–39.0)	orl[86], kem[1]	2016–2023
	211t	3	1	2	2	1	0	0	0	0	0	24.0 (24.0–32.0)	kra[3]	2014–2017
	5t	3	2	1	2	0	0		0	0	1	36.0 (31.0–41.0)	orl[3]	2015–2021
	19t	3	2	1	1	1	0	0	0	1	0	32.0 (32.0–58.0)	kya[2], bur[1]	2014–2017
	24q	4	2	2	3	1	0	0	0	0	0	37.0 (28.5–38.5)	orl[4]	2018
	229l7	7	5	2	2	5	0	0	0	0	0	29.0 (26.0–33.0)	jew[7]	2015–2017
	467t	3	3	0	0	1	0	0	0	2	0	28.0 (21.0–43.0)	orl[3]	2022
	63l6	6	4	2	2	0	0	0	3	1	0	20.5 (2.0–45.0)	sta[6]	2018
	14l238	238	150	88	84	54	0	6	2	64	28	35.0 (27.0–41.0)	alt[89],amu[1], bur[1], cha[1], irk[3], kab[1], kao[17], kem[16], kha[1], khk[1], kir[1], kra[20], kug[1], kur[2], kya[8], mag[1], mos[5], mow[2], nvs[33], oms[12], orl[3], ros[1], sak[1], spb[1], sta[6], sve[2], yan[1], zab[1], tom[6]	2011–2023
	Dyads	30	15	15	18	5	0	1	0	1	5	35.0 (29.0–43.0)	alt[10], kao[6], kra[6], mor[1], nvs[2], orl[4], tam[1]	2011–2023
	Singletons	363	211	152	136	95	5	11	1	69	46	35.0 (29.0–41.0)	alt[127], amu[2], bry[1], che[6], ing[2], jew[16], kab[1], kao[12], kem[19], kha[3], khk[14], kra[22], kug[1], kur[1], kya[2], len[1], lip[2], mag[2], mor[1], mos[6], mow[3], nvs[14], oms[14], ore[1], orl[22], pri[3], ros[1], sah[2], spb[17], sta[5], sve[1], tul[1], tyu[1], tom[3],vor[3], yan[2], yar[6]	2008–2024
	Overall	747	453	294	281	213	5	18	6	140	84	35.0 (28.0–41.0)	-	2008–2024
B	11l9	9	7	2	2	0	5	0	0	1	1	29.0 (26.0–35.0)	kra[2], len[1], mos[2], mow[2], ros[1], spb[1]	2006–2019
	132l6	6	6	0	0	1	4	0	0	1	0	36.0 (27.0–43.0)	len[4], mos[1], sam[1]	2007–2016
	135t	3	3	0	0	0	2	0	0	1	0	31.0 (20.0–35.0)	len[2], mos[1]	2007–2009
	139t	3	3	0	0	0	3	0	0	0	0	23.0 (17.0–29.0)	len[3]	2006–2010
	196l13	13	8	5	5	6	0	1	0	1	0	26.0 (18.0–37.0)	alt[1], lip[2], pri[9], spb[1]	2012–2023
	1t	3	2	1	2	0	0	0	0	0	1	40.0 (33.0–63.0)	vor[1], kao[2]	2016–2023
	20q	4	1	3	3	0	1	0	0	0	0	28.0 (26.0–36.5)	kra[4]	2015–2017
	23q	4	4	0	2	0	1	0	0	0	1	38.5 (36.5–40.0)	mow[1], cha[1], kra[2]	2008–2023
	24l5	5	4	0	2	0	2	0	0	1	0	33.0 (27.0–40.0)	kra[4], sta[1]	2014–2023
	277t	3	3	0	2	0	1	0	0	0	0	34.0 (24.0–43.0)	mos[1], mow[1], cha[1]	2019–2023
	280q	4	4	0	1	0	3	0	0	0	0	23.0 (21.5–27.0)	kra[4]	2014–2018
	297l7	7	7	0	0	0	4	0	0	2	1	26.0 (23.0–29.0)	mos[2], mow[3], sve[2]	2017–2020
	298l7	7	7	0	3	0	4	0	0	0	0	26.0 (23.0–33.0)	kra[7]	2017–2019
	309l6	6	6	0	2	0	4	0	0	0	0	29.5 (26.0–34.0)	mos[1],mow[4],cha[1]	2015–2023
	31l7	7	7	0	3	0	3	0	0	0	1	23.0 (21.0–33.0)	kra[5], mos[1], smo[1]	2015–2020
	322q	4	4	0	3	0	0	0	0	1	0	26.0 (23.5–29.5)	kag[1], kya[2], kra[1]	2012–2018
	85l5	5	5	0	1	0	3	0	0	1	0	39.0 (38.0–41.0)	pnz[1], yar[1], vlg[2], mos[1]	2014–2021
	Dyads	58	50	8	19	3	20	0	0	9	7	29.5 (23.0–39.0)	bry[2], cha[1], chv[1], iva[1], jew[2], kra[11], kur[1], kya[1], mos[10], mow[9], niz[3], orl[3], rya[1], spb[5], sve[2], tul[1], udm[1], yan[1], yar[2]	2007–2023
	Singletons	380	336	44	119	28	142	3	0	42	46	31.0 (25.0–38.0)	alt[1], amu[1], ast[1], bry[5], cha[4], che[6], dag[1], ing[1], iva[1], jew[3], kag[1], kai[1], kal[4], kar[1], kha[1], khm[1], kir[1], kos[3], kra[24], kug[1], len[26], lip[5], mag[1], mor[1], mos[89], mow[44], mur[2], niz[11], nor[1], nvs[3],orl[15], pnz[1], pri[18], psk[1], rya[4], sak[1], sam[1], sar[1], smo[7], spb[42], sta[4], sve[2], tam[2], tat[1], tul[8],tve[4], tyu[3], udm[1], vgg[3], vlg[2], vor[4], yan[6], yar[3]	1995–2024
	Overall	531	467	63	169	38	202	4	0	60	58	30.0 (25.0–38.0)	-	1995–2024

02_AG$_{FSU}$	16q	4	4	0	0	0	3	0	0	1	0	27.5 (27.0–28.5)	kem^{1}, mow^{3}	2018–2022
	20t	3	1	2	0	3	0	0	0	0	0	30.0 (29.0–34.0)	lip^{3}	2015–2022
	2t	3	2	1	2	1	0	0	0	0	0	44.0 (17.0–52.0)	sam^{1}, pnz^{1}, mos1	2015–2018
	3t	3	3	0	0	0	2	0	0	1	0	32.0 (32.0–38.0)	kra^{2}, ros^{1}	2017–2018
	4t	3	2	1	0	0	2	0	0	1	0	28.0 (22.0–30.0)	mow^{2}, irk^{1}	2014–2023
	51t	3	1	2	0	3	0	0	0	0	0	30.0 (27.0–37.0)	mos^{3}	2019–2020
	Dyads	16	7	9	7	0	2	0	0	7	0	32.5 (26.0–42.0)	bry^{1}, kao^{2}, kra^{1}, lip^{2}, mos^{7}, orl^{2}, spb^{1}	2012–2023
	Singletons	76	50	26	36	10	12	3	0	8	7	32.0 (24.0–40.0)	alt^{2}, amu^{1}, bry^{2}, che^{2}, kab^{1}, kal^{2}, kao^{1}, kos^{1}, kra^{3}, kur^{1}, lip^{4}, mor^{1}, mos^{16}, mow^{9}, mur^{2}, niz^{1}, ore^{2}, orl^{3}, per^{1}, pri^{1}, ros^{2}, sah^{1}, sam^{6}, spb^{2}, sve^{1}, tul^{3}, tve^{1}, vor^{4}	2007–2023
	Overall	111	70	41	45	17	21	3	0	18	7	32.0 (25.0–39.0)	-	2007–2023
03_A6B	5l20	20	7	13	10	5	0	1	0	4	0	23.0 (21.0–31.5)	vgg^{1}, kao^{1}, kra^{1}, spb^{1}, mos^{2}, sve^{12}, yan^{1}, che^{1}	2007–2023
	21t	3	0	3	0	0	0	0	0	3	0	35.0 (32.0–39.0)	sve^{3}	2011–2012
	Dyads	2	2	0	1	0	1	0	0	0	0	47.0 (47.0–47.0)	lip^{2}	2012
	Singletons	43	17	26	16	7	0	2	0	15	3	26.0 (22.0–33.0)	che^{2}, kag^{5}, kra^{1}, kug^{1}, len^{1}, ngr^{1}, nvs^{1}, orl^{1}, sak^{2}, smo^{1}, spb^{1}, sta^{1}, sve^{15}, tyu^{3}, vlg^{2}, vor^{1}, yan^{3}, yar^{1}	2008–2023
	Overall	68	26	42	27	12	1	3	0	22	3	26.0 (22.0–34.0)	-	2007–2023
14/73_BG	17l9	9	8	1	3	0	3	0	1	0	2	27.0 (26.0–34.0)	dag^{1}, kal^{1}, kra^{3}, mos^{1}, mow^{1}, yar^{2}	2015–2023
	8l7	7	7	0	4	0	3	0	0	0	0	25.0 (24.0–28.0)	kab^{1}, kra^{3}, mos^{1}, mow^{2}	2010–2021
	Dyads	10	10	0	5	1	2	0	0	2	0	28.5 (22.0–32.0)	bas^{2}, kao^{1}, kra^{3}, mos^{2}, mow^{2}	2008–2023
	Singletons	33	28	5	13	1	14	0	0	5	0	30.0 (25.0–40.0)	alt^{1}, kir^{1}, mos^{7}, mow^{7}, spb^{2}, tat^{10}, tul^{2}, tve^{2}, vlg^{1}	2009–2023
	Overall	59	53	6	25	2	22	0	1	7	2	28.0 (24.0–38.0)	-	2008–2023

[1] The upper index preceding the 3-letter code for a subject indicate the number of sequences from this subject. IQR, interquartile range; HET, heterosexual contacts; IDUs, injecting drug users; MSM, men who have sex with men; MTCT, mother-to-child transmission; NSC, nosocomial transmission; UNK, unknown; ST, sexual transmission; IQR, interquartile range. 3-letter code list: alt, altai krai; amu, amur oblast; ark, arkhangelsk oblast; ast, astrakhan oblast; bel, belgorod oblast; bry, bryansk oblast; vla, vladimir oblast; vgg, volgograd oblast; vlg, vologda oblast; vor, voronezh oblast; mow, federal city of moscow; spb, federal city of saint petersburg; sev, federal city of sevastopol; jew, jewish autonomous oblast; zab, zabaykalsky krai; iva, ivanovo oblast; irk, irkutsk oblast; kab, kabardino-balkar republic; kag, kaliningrad oblast; kal, kaluga oblast; kam, kamchatka krai; kao, karachay-cherkess republic; kem, kemerovo oblast; kir, kirov oblast; kos, kostroma oblast; kra, krasnodar krai; kya, krasnoyarsk krai; kug, kurgan oblast; kur, kursk oblast; len, leningrad oblast; lip, lipetsk oblast; mag, magadan oblast; mos, moscow oblast; mur, murmansk oblast; nen, nenets autonomous okrug; niz, nizhny novgorod oblast; ngr, novgorod oblast; nvs, novosibirsk oblast; oms, omsk oblast; ore, orenburg oblast; orl, oryol oblast; pnz, penza oblast; per, perm krai; pri, primorsky krai; psk, pskov oblast; ady, republic of adygea; ali,altai republic; bas, republic of bashkortostan; bur, republic of buryatia; dag, republic of dagestan; ing, republic of ingushetia; kai, republic of kalmykia; kar, republic of karelia; kom, komi republic; crm, republic of crimea; mar, mari el republic; mor, republic of mordovia; sah, sakha republic; nor, republic of north ossetia-alania; tat, republic of tatarstan; tuv, tuva republic; khk, republic of khakassia; ros, rostov oblast; rya, ryazan oblast; sam, samara oblast; sar, saratov oblast; sak, sakhalin oblast; sve, sverdlovsk oblast; smo, smolensk oblast; sta, stavropol krai; tam, tambov oblast; tve, tver oblast; tom, tomsk oblast; tul, tula oblast; tyu, tyumen oblast; udm, udmurt republic; uly, ulyanovsk oblast; kha, khabarovsk krai; khm, khanty-mansi autonomous okrug; che, chelyabinsk oblast; cha, chechen republic; chv, chuvash republic; chu, chukotka autonomous okrug; yan, yamalo-nenets autonomous okrug; yar, yaroslavl oblast.

Table S4. Factors associated with high linkage (≥4 link) in the transmission networks.

Attribute	Category	Total, *N*	Individuals with Low Linkage (<4 link), *N* (%)	Individuals with High Linkage (≥4 Link), *N* (%)	Adjusted Odds Ratio (95% CI)	*p*-Value
Age at diagnosis (years)	≥50	117	88 (75.2)	29 (24.8)	Ref	
	30–49	866	595 (68.7)	271 (31.3)	1.19 (0.74–1.90)	0.463
	<30	861	582 (67.6)	279 (32.4)	0.85 (0.52–1.39)	0.524
Sex	Female	733	526 (71.8)	207 (28.2)	Ref	
	Male	1111	739 (66.5)	372 (33.5)	1.20 (0.9–1.50)	0.097
Transmission risk	HET	782	605 (77.4)	177 (22.6)	Ref	
	IDUs	525	276 (52.6)	249 (47.4)	3.05 (2.31–4.03)	<0.001
	MSM	137	120 (87.6)	17 (12.4)	0.47 (0.25–0.86)	0.149
	MTCT	52	40 (76.9)	12 (23.1)	1.36 (0.64–2.89)	0.423
	NSC	26	23 (88.5)	3 (11.5)	0.44 (0.13–1.57)	0.208
	UNK	225	139 (61.8)	86 (38.2)	1.60 (1.11–2.30)	0.011
	ST	97	62 (63.9)	35 (36.1)	2.17 (1.27–3.71)	0.005
ART status	Treated	670	458 (68.4)	212 (31.6)	Ref	
	Naive	1174	807 (68.7)	367 (31.3)	1.26 (1.00–1.58)	0.045
HIV-1 subtype	A6	1223	921 (75.3)	302 (24.7)	Ref	
	B	151	127 (84.1)	24 (15.9)	0.58 (0.36–0.93)	0.025
	63_02A6	384	149 (38.8)	235 (61.2)	7.36 (5.57–9.71)	<0.001
	02_AG_{FSU}	35	35 (100.0)	0 (0)	N/d	
	03_A6B	25	11 (44)	14 (56)	3.66 (1.59–8.40)	0.002
	14/73_BG	26	22 (84.6)	4 (15.4)	0.69 (0.23–2.10)	0.517
Region of sampling [1]	CN	783	554 (70.8)	229 (29.2)	Ref	
	FE	80	57 (71.3)	23 (28.8)	0.79 (0.46–1.35)	0.394
	NC	85	60 (70.6)	25 (29.4)	0.76 (0.45–1.30)	0.325
	NW	47	44 (93.6)	3 (6.4)	0.13 (0.04–0.42)	0.001
	SB	317	176 (55.5)	141 (44.5)	1.29 (0.96–1.75)	0.094
	ST	294	226 (76.9)	68 (23.1)	0.73 (0.53–1.02)	0.063
	UR	97	60 (61.9)	37 (38.1)	1.12 (0.69–1.81)	0.638
	VL	141	88 (62.4)	53 (37.6)	1.21 (0.80–1.83)	0.372
Diagnosis date	1988–2002	284	146 (51.4)	138 (48.6)	Ref	
	2003–2015	727	521 (71.7)	206 (28.3)	0.32 (0.23–0.44)	<0.001
	2015–2023	833	598 (71.8)	235 (28.2)	0.22 (0.15–0.32)	<0.001

[1] Federal District of Russia. CI, confidence interval; HET, heterosexual contacts; IDUs, injecting drug users; MSM, men who have sex with men; MTCT, mother-to-child transmission; NSC, nosocomial transmission; UNK, unknown; ST, sexual transmission; IQR, interquartile range; CN, Central FD; FE, Far East FD; NC, North Caucasian FD; NW, Northwestern FD; SB, Siberian FD; ST, Southern FD; UR, Ural FD; VL, Volga FD.

Table S5. The share of inferred viral migration events from each source location for six major HIV-1 subtypes in Russia.

Location [1]	Proportion of Viral Transmissions from Region, %
Chelyabinsk oblast (CHE)	26.3
Krasnodar Krai (KRA)	24.5
Krasnoyarsk Krai (KYA)	47.1
City of Moscow (MOW) + Moscow Oblast (MOS)	78.7
Nizhny Novgorod Oblast (NIZ)	36.7
Oryol Oblast (ORL)	39.7
Samara Oblast (SAM)	42.3
City of Saint Petersburg (SPB) + Leningrad oblast (LEN)	45.8
Tver Oblast (TVE)	66.1
Tula Oblast (TUL)	51.9

[1] Only locations accounting for >2.0% of all sequences within a total dataset are described in the table.
